# Regulation of Autophagy by the Glycogen Synthase Kinase-3 (GSK-3) Signaling Pathway

**DOI:** 10.3390/ijms23031709

**Published:** 2022-02-01

**Authors:** Hsuan-Yeh Pan, Mallika Valapala

**Affiliations:** School of Optometry, Indiana University, Bloomington, IN 47405, USA; hsupan@iu.edu

**Keywords:** mTORC1, TFEB, GSK-3, GSK-3β, autophagy, lysosome, AKT, PKC, ULK1

## Abstract

Autophagy is a vital cellular mechanism that benefits cellular maintenance and survival during cell stress. It can eliminate damaged or long-lived organelles and improperly folded proteins to maintain cellular homeostasis, development, and differentiation. Impaired autophagy is associated with several diseases such as cancer, neurodegenerative diseases, and age-related macular degeneration (AMD). Several signaling pathways are associated with the regulation of the autophagy pathway. The glycogen synthase kinase-3 signaling pathway was reported to regulate the autophagy pathway. In this review, we will discuss the mechanisms by which the GSK-3 signaling pathway regulates autophagy. Autophagy and lysosomal function are regulated by transcription factor EB (TFEB). GSK-3 was shown to be involved in the regulation of TFEB nuclear expression in an mTORC1-dependent manner. In addition to mTORC1, GSK-3β also regulates TFEB via the protein kinase C (PKC) and the eukaryotic translation initiation factor 4A-3 (eIF4A3) signaling pathways. In addition to TFEB, we will also discuss the mechanisms by which the GSK-3 signaling pathway regulates autophagy by modulating other signaling molecules and autophagy inducers including, mTORC1, AKT and ULK1. In summary, this review provides a comprehensive understanding of the role of the GSK-3 signaling pathway in the regulation of autophagy.

## 1. Introduction

Glycogen synthase kinase-3 (GSK-3) has been identified to play a vital role in glucose metabolism [1]. Glycogen synthase kinase-3 (GSK-3) has two different paralogs, GSK-3α and GSK-3β [2,3]. GSK-3 is a serine/threonine kinase and was shown to suppress the activation of glycogen synthase, an important enzyme in glycogen metabolism [1]. The activation of GSK-3β is determined by the phosphorylation and dephosphorylation of GSK-3β on several sites [3,4]. Autophosphorylation at Tyr216 is known to constitutively activate GSK-3β and Tyr216 is also known to be phosphorylated by Src, a non-receptor tyrosine kinase [5,6]. Protein kinase B (AKT), protein kinase C (PKC) and the p90RSK signaling pathways are known to be involved in the phosphorylation of GSK-3β at serine 9 (Ser9) and its regulation [5].

Several transcription factors in the nucleus were identified as being regulated by GSK-3β. Cellular Myc (c-Myc) at threonine 58 (Thr58) and Snail at serine 96 (Ser96) and serine 100 (Ser100) can be phosphorylated by GSK-3β and trigger ubiquitin-dependent proteasomal degradation of c-Myc and Snail [4,7,8,9]. Inhibition of mTORC1 was shown to stimulate GSK-3β nuclear localization [4,10]. Studies have shown that GSK-3β nuclear localization can be induced by inhibition of mTORC1 using mTORC1 inhibitor rapamycin, resulting in the reduction of c-Myc and Snail [4,10]. Inhibition of GSK-3β is also known to enhance autophagy and lysosomal acidification [11,12]. GSK-3β is known to be involved in neuronal development and suppression of GSK-3β showed the ability to reduce α-synuclein in cellular models of Parkinson’s disease [2,13,14,15]. GSK-3β was also reported to be associated with amyotrophic lateral sclerosis (ALS), elevated levels of active GSK-3β were found in the brain of ALS patients [2]. Inhibition of GSK-3β using lithium was shown to slow down the progression of ALS in ALS patients [2,16].

The mammalian target of rapamycin (mTOR) is a protein kinase comprised of two different complexes, mammalian target of rapamycin complex 1 (mTORC1) and mammalian target of rapamycin complex 2 (mTORC2) [17,18]. mTORC1 and mTORC2 have different components, structure, and functions [17,18]. mTORC1 and mTORC2 also show a difference in sensitivity to rapamycin; mTORC1 is highly responsive to rapamycin compared to mTORC2 which is not responsive to rapamycin [19]. mTORC1 complex contains several proteins that regulate its activity [20]. mTORC1 translocates to the lysosomal surface and is activated in an amino acid-rich environment [20]. Multiple studies have shown that mTORC1 plays a crucial role in the regulation of the autophagy pathway [19,21]. Inhibition of mTORC1 was shown to upregulate autophagy [19,21]. In the absence of amino acids, mTORC1 is translocated from the lysosomal surface to the cytosol [21]. In the presence of amino acids, Rag GTPases are activated, which are involved in the transportation of mTORC1 to the surface of the lysosomes [22,23]. Ragulator activates Rag GTPases in an amino acid-dependent manner and facilitates the interaction of Rag GTPases with Raptor, one of the components of mTORC1 complex [24]. The interaction of Rag GTPase with Raptor can induce mTORC1 transportation from cytosol to lysosomal surface, where Ras homolog enriched in brain (Rheb) switches mTORC1 into an active form [23,24]. The functions of mTORC1 are mainly associated with protein synthesis and autophagy [24]. mTORC1 regulates cellular functions by regulating the expression of downstream targets, ribosomal S6 kinases (S6K) and eukaryotic translation initiation factor 4E (eIF4E]-binding protein 1 (4E-BP1) [24,25,26]. 4E-BP1 inhibits translation initiation via binding to the eukaryotic initiation factor 4E (eIF4E) and prevents the interaction of eIF4E and other eukaryotic initiation factors [24,26]. Activated mTORC1 can phosphorylate 4E-BP1 resulting in the dissociation of 4E-BP1 from eIF4E and interaction of elF4E with elF4G leading to translation initiation [24,26]. Activated mTORC1 also phosphorylates S6K at threonine 389 (Thr389) and activated S6K enhances protein synthesis [27]. Thus, 4E-BP1 and S6K are often described as mTOR downstream mediators. mTORC1 was also reported to regulate mitochondrial function [28]. Studies have shown activation of mTOR in mouse models of mitochondrial myopathy (MM) [29]. MM causes respiratory chain (RC) dysfunction due to mutation of mitochondrial replicative helicase twinkle [29]. mTORC1 has shown to be responsible for several stress responses caused by mitochondrial dysfunction in mouse models of MM [29]. mTORC1 inhibitor rapamycin was shown to alleviate mitochondrial unfolded protein response and other stress responses to reverse the progression of MM [29].

Autophagy is a cellular degradation process that delivers intercellular cargo to the lysosomes [30]. Autophagy can be triggered by cellular stress including nutrient deprivation and hypoxia [31]. Furthermore, autophagy can degrade long-lived proteins and organelles and recycle them to support cell survival [32]. The process of autophagy involves several steps including autophagy induction, autophagosome formation, and the fusion of autophagosome and lysosomes [33]. UNC-51-like kinase (ULK1) is a serine/threonine kinase and was identified as one of the components to initiate the autophagy pathway [34]. The activation of mTORC1 was shown to inhibit ULK1 activity [35]. Inhibition of mTORC1 results in a promotion of ULK1 activity followed by autophagosome formation and induction of the autophagy pathway [34,35]. Autophagosome is a double-membrane vesicle containing intercellular material [36]. In the last steps of the autophagy pathway, autophagosomes fuse with lysosomes to form autolysosomes for the degradation of cellular material that is inside the autophagolysosome [37]. Inhibition of mTORC1 was shown to enhance autophagy pathway [38,39]. Studies have shown that repression of mTORC1 by hayatine, an mTORC1 inhibitor, can enhance autophagy flux in tumor cells [39]. Other studies have shown that activation of mTORC1 by palmitic acid (PA) impaired autophagy flux and repressed lysosomal associated membrane protein-2 (LAMP2) expression in mouse Hepa-1c1c7 cells [40]. Furthermore, mTORC1 inhibition by rapamycin showed an induction of autophagy in C57BL/6J mice fed with an alcohol-containing diet for 8 weeks [40]. mTORC1 hyperactivation was reported in the isolated islet of type 2 diabetes patients and type 2 diabetes animal models [41]. Impaired autophagy is one of the features of type 2 diabetes, mTORC1 hyperactivation induced by large-tumor suppressor 2 (LATS2), suppressed autophagy and further triggered pancreatic β-cell apoptosis [42]. The activation of mTORC1 was shown to be potentially associated with RPE degeneration in the pathogenesis of age-related macular degeneration (AMD) [43,44,45]. A study has shown that upregulation of ULK1 and 4E-BP1 phosphorylation is observed in RPE/choroid tissues from dry AMD patients [43]. These findings also suggest that hyper activation of the mTORC1 pathway is associated with the suppression of autophagy in dry AMD patients [43]. Studies have also shown activated mTORC1 results in RPE degeneration [44,45]. In this review, we will discuss the mechanisms by which the GSK-3 signaling pathway regulates autophagy in an mTORC1-dependent and independent manner.

## 2. GSK-3-Mediated Regulation of TFEB

**Figure 1:**
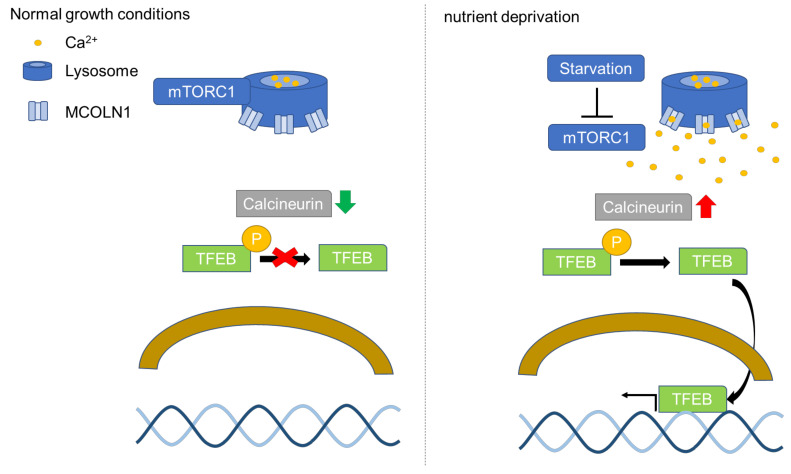
The regulation of TFEB by mTORC1. MCOLN1(TRPML1) at serine 572 (Ser572) and serine 576 (Ser576) can be phosphorylated by activated mTORC1 located on the lysosomal surface to suppress MCOLN1 activity [56]. However, the inhibition of mTORC1 via starvation activates MCOLN1 and release calcium from lysosomes [52,57]. The increase in calcium in cytosol activates calcineurin and further leads to calcineurin-mediated dephosphorylation of TFEB. Dephosphorylated TFEB translocates to the nucleus to activate gene expression of CLEAR network genes to promote lysosomal biogenesis and autophagy.

Transcriptional factor EB (TFEB) is identified as an activator of autophagy through the upregulation of autophagy and lysosomal genes [46]. TFEB-regulated downstream genes are involved in different steps of the autophagy pathway, including autophagy initiation, autophagosome formation, and the fusion of autophagosome and lysosome [46]. Furthermore, TFEB in the nucleus activates the expression of genes in the coordinated lysosomal expression and regulation (CLEAR) network to enhance autophagy and lysosomal biogenesis [46,47]. TFEB is a transcription factor belonging to the MITF/TFE family [46]. The subcellular localization of TFEB is regulated by mTORC1 [46]. Activated mTORC1 phosphorylates two residues of TFEB, serine 142 (Ser142) and serine 211 (Ser211), leading to TFEB cytosolic retention and further inhibiting TFEB activation [48]. TFEB in the cytosol can be translocated to the lysosomal surface to be phosphorylated by mTORC1, leading to cytosolic retention of TFEB [49]. The activation of mTORC1 phosphorylates TFEB at Ser211 and promotes TFEB binding to the 14-3-3 protein, which masks the nuclear localization signal (NLS) and prevents TFEB nuclear localization [50]. TFEB can be dephosphorylated by calcineurin, a phosphatase resulting in nuclear translocation of TFEB [51]. TFEB nuclear translocation was shown to be induced by constitutively active calcineurin in cells grown in a nutrient-rich environment [52]. The activation of calcineurin is correlated with the concentration of calcium in the cytosol of cells [53]. Mucolipin 1 (MCOLN1), also known as TRPML1, is a calcium channel on the lysosomal membrane and activation of MCOLN1 triggers calcium release from the lysosomes and enhances the activation of calcineurin [54,55]. mTORC1 phosphorylates MCOLN1 at serine 572 (Ser572) and serine 576 (Ser576) on the lysosomal surface to inactivate MCOLN1 [56]. Nutrient deprivation in cells results in mTORC1 dissociation from the lysosomes and mTORC1 is no longer able to phosphorylate MCOLN1 [52,56]. Activated calcineurin dephosphorylates Ser142 and Ser211 of TFEB and triggers TFEB nuclear translocation (Figure 1) [55]. 

### 2.1. Regulation of TFEB by the mTORC1-GSK-3β Signaling Pathway

mTORC1 is known to inactivate GSK-3β through ribosomal protein S6 kinase beta-1 (S6K1), a downstream substrate of mTORC1 [58,59]. Studies have shown that mTORC1 inhibition decreases the phosphorylation of GSK-3β, resulting in increased activation of GSK-3β [59]. p85S6K, one of S6K1 isoforms, was shown to phosphorylate GSK-3β at Ser9 and leads to the inactivation of GSK-3β [59]. Furthermore, inhibition of p85S6K by S6K1 inhibitor abolished the phosphorylation of GSK-3β at Ser9 [59].

**Figure 2:**
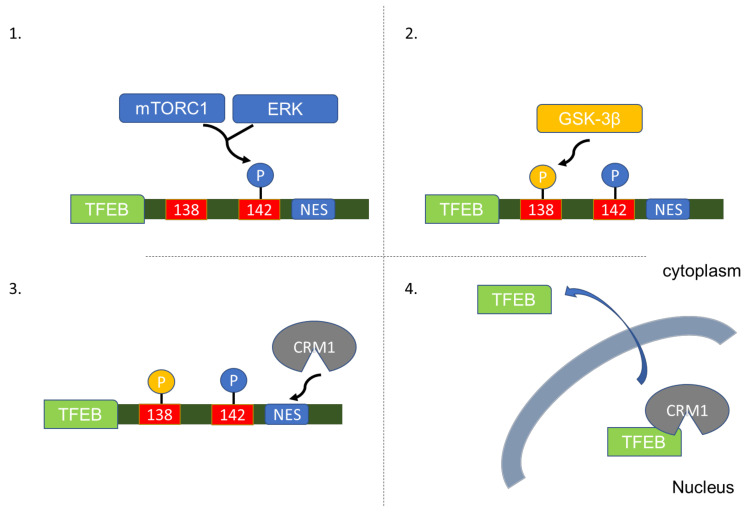
Regulation of TFEB nuclear export by mTORC1 and GSK-3β. Studies have shown that phosphorylation of TFEB at ser142 by mTORC1 and ERK primes TFEB for phosphorylation at ser138 by GSK-3β (panels 1–2) [60,61]. Phosphorylation of TFEB at ser142 and ser138 facilitates the interaction of CRM1 and TFEB (panel 3), which leads to the translocation of TFEB from nucleus to cytosol (panel 4) [61].

GSK-3β was reported to phosphorylate TFEB at serine 138 (Ser138) and facilitate TFEB nuclear export [60]. Phosphorylation of TFEB at Ser138 by GSK-3 was shown to depend on the activation of mTORC1 [61]. Studies have suggested an involvement of GSK-3β-mTORC1 and ERK signaling pathways in the nuclear export of TFEB [60,61]. mTORC1 and the MAPK extracellular signal-regulated kinase (ERK) were reported to phosphorylate TFEB at Ser142 [60,61]. Phosphorylation of TFEB at Ser142 stimulate GSK-3β-mediated phosphorylation of TFEB at Ser138 [60,61]. Studies have shown that mTORC1 regulates TFEB subcellular localization from the nucleus to cytoplasm in a chromosomal maintenance 1 (CRM1)-dependent manner in HeLa cells [61]. The nuclear export signal (NES) is recognized by CRM1, also known as Exportin-1 (XPO1), which is known to regulate the nuclear export of several proteins including TFEB [62,63]. The inhibition of CRM1 prevents nuclear export of TFEB and enhances TFEB levels in the nucleus and induces autophagy flux in vitro in rat primary cortical neurons and HeLa cells [62,63]. TFEB phosphorylation at Ser142 and Ser138 are required for TFEB translocation from nucleus to cytoplasm [60,61]. These residues are in close proximity to the NES of TFEB and possibly involved in the binding of CRM1 to the NES site of TFEB and facilitate TFEB nuclear export [60,61,64]. Mutation of Ser142 and Ser138 to alanine was shown to suppress the nuclear export of TFEB, leading to TFEB nuclear retention [60,61]. Mutation of Ser138 only affected the ability of GSK-3 to phosphorylate TFEB at Ser138 but not Ser142 [61]. These studies implied that TFEB and CRM1 interaction possibly requires Ser142 and Ser138 phosphorylation, suggesting mTORC1 and GSK-3 affect the interaction of TFEB and CRM1 [61]. Inhibition of mTORC1 by Torin was also shown to abrogate phosphorylation of nuclear TFEB at Ser138 and Ser142 [61]. These suggest that GSK-3 regulates TFEB phosphorylation and subcellular localization in an mTORC1-dependent manner (Figure 2).

### 2.2. Regulation of TFEB by PKC-GSK-3β Signaling Pathway

In addition to mTORC1, GSK-3β is also reported to be regulated by other upstream targets that in turn regulate TFEB nuclear export [65,66]. Protein kinase C (PKC) is serine/threonine protein kinases and was reported to regulate autophagy [67]. The phosphorylation of ULK1 at serine 423 (Ser423) by protein kinase C alpha (PKCα) was shown to prevent ULK1 and Syntaxin 17 (STX17) interaction leading to inhibition of autolysosome formation [67]. Moreover, a study showed that the activation of PKCα and δ can suppress GSK-3β, resulting in repression of TFEB phosphorylation at Ser134 and Ser138 and an increase in TFEB nuclear localization and activation [65]. Furthermore, HEP14 (5β-O-angelate-20-deoxyingenol), a compound that can induce the activity of PKCα and protein kinase C delta (PKCδ), was used to enhance PKCα and PKCδ activity and further triggered TFEB activation [65]. The activation of mTORC1 was not affected by the HEP14 treatment, which indicated that PKCα and δ-mediated TFEB nuclear localization occurs through GSK-3β in an mTORC1-independent manner [65].

### 2.3. Regulation of TFEB by elF4A3-GSK-3β Signaling Pathway

The expression of GSK-3β was downregulated by depletion of eukaryotic translation initiation factor 4A-3 (eIF4A3) [66]. eLF4A3 was identified as a component of the exon junction complex, which has several functions including mRNA export and splicing of mRNA [68,69]. Thus, eIF4A3 also plays a key role in regulating mRNA splicing and mRNA quality control [69]. Reduction of eIF4A3 was shown to induce TFEB nuclear localization and further leads to enhanced autophagy flux [66]. Suppression of eIF4A3 resulted in exon-skipping of GSK-3β, which leads to suppression of GSK-3β activity [66]. Inhibition of GSK-3β has been known to suppress phosphorylation of TFEB [65]. In order to examine whether GSK-3β is involved in the nuclear translocation of TFEB induced by suppression of eIF4A3, cells that overexpressed GSK-3β were co-treated with eIF4A3 siRNA and showed abrogation of TFEB nuclear translocation suggesting the role of GSK-3β in the eIF4A3-TFEB pathway [66]. 

## 3. Regulation of Autophagy by AKT-GSK-3β Signaling Pathway

Protein kinase B (AKT) is known to be involved in several signaling pathways and cellular functions including apoptosis and gene transcription [70]. More than 50 proteins were identified as being regulated by AKT, including mTORC1 and GSK-3 [71,72]. AKT phosphorylation is key to the regulation of AKT activity, for example, GSK-3α phosphorylates AKT at threonine 312 to inactivate AKT [72,73]. The phosphoinositide-dependent protein kinase 1 (PDK1) and mTORC2 were identified to phosphorylate AKT and trigger AKT activity [72]. AKT is also identified to modulate TFEB by phosphorylating TFEB at serine 467 (Ser467), leading to suppression of TFEB nuclear localization [74]. Inhibition of AKT using AKT inhibitor, trehalose and MK-2206, showed induction of TFEB nuclear localization in both WT and lysosomal associated membrane protein-2 (LAMP2) knockout (KO) mouse RPE cells [75]. Furthermore, oral trehalose administration induced the expression of CLEAR genes and stimulated TFEB nuclear localization [74]. Trehalose also showed an increase in the LC3-II/I ratio in WT and LMP2 KO RPE [75]. The induction of autophagy by AKT inhibitor, trehalose, was shown to rescue several disease phenotypes in cell and animal models [76,77]. Exposure to cigarette smoke or hydroquinone, present in cigarette smoke, result in oxidative damage to the RPE [76]. The study shows that oxidative damage induced by hydroquinone can be inhibited by upregulation of TFEB and CLEAR network gene expression by AKT inhibitor, trehalose [76]. Other studies have also shown induction of LC3 II expression in trehalose treatment, suggesting autophagy was activated by trehalose in the acute kidney injury (AKI) mouse model [77]. Additionally, the mitochondrial dysfunction and fragmentation in AKI mice were also shown to be rescued by trehalose [77]. The AKT signaling pathway is known to phosphorylate GSK-3β at Ser9 to inactivate GSK-3β [78]. Interestingly, knockdown of GSK-3β also suppresses AKT and further stimulated autophagy [79]. It was shown that repression of GSK-3β decreased AKT activity and enhanced AMPK activity, leading to the induction of forkhead box protein O1 (FOXO1) [79]. FOXO1 is a transcription factor, which plays an important role in the induction of autophagosome formation in Human Aortic Endothelial Cells (HAECs) [79]. Furthermore, this study showed that the activity of mTORC1 was not altered in GSK-3β knockdown HAECs, suggesting suppression of GSK-3β induces autophagy in an mTORC1-independent manner in specific cell types [79]. 

## 4. GSK-3β-Mediated Regulation of ULK1

ULK1 is one of the downstream targets of mTORC1 and plays a crucial role in the initiation of the autophagy pathway [34,80]. The activation of ULK1 is controlled by phosphorylation and dephosphorylation [81]. ULK1 is part of the ULK1 complex formed with autophagy-related protein 101 (ATG101), autophagy-related protein 13 (ATG13), and focal adhesion kinase family interacting protein of 200 kD (FIP200) in the cells [34,81]. The activation of ULK1 is regulated by mTORC1. mTORC1 phosphorylates ULK1 and suppresses its catalytic activity [34,80,81]. It was reported that mTORC1 phosphorylates ULK1 at serine 757 (Ser757) and inhibits its activity in nutrient-rich conditions [35,38]. The inactive form of mTORC1 in starvation and cellular stress can dissociate from the ULK1 complex [38]. Thus, ULK1 can be dephosphorylated by protein phosphatase 2A (PP2A) and protein phosphatase 1D magnesium-dependent delta isoform (PPM1D) [38,82]. Autophosphorylation at Thr180 of ULK1 triggers ULK1 activation [38,82]. Inhibition of mTORC1 can enhance ULK1 activity to trigger the initiation of autophagy by phosphorylating the autophagy-associated downstream targets of ULK1 including autophagy-related protein 9 (ATG9) and Beclin 1 (BECN1) [83]. ATG9 functions as a transmembrane protein and participates in autophagosome formation [84,85]. An ATG9 vesicle formed by ATG9 is mobilized to the pre-autophagosomal structure (PAS) in starvation conditions and functions as a seed to the growing phagophore (also known as isolation membrane), which can be expanded to form the autophagosome [84,85]. BECN1 forms a class III phosphatidylinositol 3-kinase (PI3K-III) complex by interacting with VPS_34_ and other factors for autophagosome formation [86]. Furthermore, it was reported that ULK1 can phosphorylate BECN1 at serine 15 (Ser15) and serine 30 (Ser30) to enhance BECN1 interaction with other autophagy-associated proteins to facilitate autophagosome maturation and autophagosome biogenesis [86]. It was also reported that ULK1 is not only involved in the steps of autophagy initiation but also in the fusion of autophagosome and lysosome [67]. Activated ULK1 interacts with Syntaxin 17 (STX17) and mobilizes STX17 to autophagosomes, which facilities the interaction between STX17 and synaptosomal-associated protein 29 (SNAP29) to form a complex [67]. This STX17 complex is known to be involved in the steps of autophagosome and lysosome fusion [87]. Inhibition of ULK1 by ULK1 inhibitor, ULK-101, was shown to suppress autophagic nucleation and autophagy flux in U2OS cells [88].

Activated GSK-3β phosphorylates ULK1 at serine 405 (Ser405) and serine 415 (Ser415) in GABA Type A Receptor-Associated Protein (GABARAP)-interacting region of ULK1, leading to the induction of autophagy in adult hippocampal neural stem (HCN) cells [89]. In addition to ULK1 phosphorylation directly by GSK-3, GSK-3 was also reported to regulate ULK1 through HIV-1 Tat interactive protein, 60 kD (TIP60), an acetyltransferase [90]. The activation of GSK-3 phosphorylates TIP60 at serine 86 (Ser86) and stimulates the activation of TIP60, which leads to acetylation of ULK1 [90]. The phosphorylation of TIP60 at Ser86 by GSK-3 can be suppressed by GSK-3 inhibitor, suggesting that GSK-3 is an upstream regulator of TIP60 [90]. The endoplasmic reticulum (ER) plays a vital role in protein synthesis and maturation, thus, endoplasmic reticulum (ER) stress is involved in the pathogenesis of several diseases [91]. ER stress was shown to induce autophagy for cell survival under cellular stress such as oxidative stress and hypoxia [91,92,93]. The activation of GSK-3β is reported to be induced by ER stress and further triggers autophagy through the GSK-3β-TIP60-ULK1 pathway [91]. The ER stress inducer, Tunicamycin (TM), was shown to decrease phosphorylation of GSK-3β at serine 9 (Ser9), which activates GSK-3β-mediated phosphorylation of TIP60 at Ser86 [91]. Phosphorylation of TIP60 by GSK-3β, in turn, leads to activation of ULK1 by acetylation resulting in an enhancement of ER stress-induced autophagy activation [91]. 

## 5. GSK-3-Mediated Regulation of the mTORC1 Signaling Pathway

GSK-3 was also reported to function upstream of mTORC1 by directly phosphorylating the regulatory associated protein of mTOR (Raptor) at serine 859 (Ser859) [94]. Inhibition of GSK-3 showed decreased phosphorylation of Raptor on Ser859, which prevents Raptor and mTOR interaction, leading to inhibition of mTORC1 [94]. GSK-3 suppression showed the ability to meditate lysosomal acidification through an mTORC1-dependent manner [12]. On the other hand, both GSK-3 inhibition and GSK-3 activation were shown to regulate mTORC1 via tuberous sclerosis complex 2 (TSC2) [12,95,96]. Inhibition of GSK-3 is shown to activate TSC2, a negative regulator of mTORC1, further leading to the repression of mTORC1 [12]. It is also shown that the effect of GSK-3 inhibition on mTORC1 was abolished in TSC knockout MEF cells [12]. TSC2, a GTPase-activating protein (GAP), regulates mTORC1 through Rheb, which converts Rheb-guanosine triphosphate (GTP) into Rheb-guanosine diphosphate (GDP) form to inactivate Rheb, leading to mTORC1 inactivation [97,98]. The inhibition of GSK-3 α/β using the CHIR99021 GSK-3 α/β inhibitor was shown to enhance the ratio of LC3 A/B-II to LC3 A/B-I and effectively decrease p62 expression in epithelioid sarcoma cells [11], suggesting that induction of autophagy occurs in epithelioid sarcoma cells [11]. Moreover, this study also examined the mTORC1 expression and found downregulation of mTOR and p-mTOR expression in CHIR99021 GSK-3 α/β inhibitor treatment [11]. On the other hand, some reports show that activation of GSK-3 phosphorylates and activates TSC2, leading to downregulation of mTORC1 [95,96]. However, TSC2 needs to be phosphorylated at serine 1345 (Ser1345) by AMPK in order for GSK-3 to phosphorylate TSC2 [96].

## 6. mTORC1 Regulates Foxk1 through GSK-3

Several studies also show that inhibition of mTORC1 regulates the transcription factor forkhead/winged-helix family k1 (Foxk1) phosphorylation through the GSK-3 signaling pathway [99,100]. Foxk1 was shown to suppress autophagy as a transcriptional repressor [101]. Foxk1 was reported to participate in several cellular mechanisms including cellular metabolism [102]. The translocation of FoxK1 from the cytoplasm to the nucleus was shown to be regulated by mTORC1 [101]. Treatment of mTORC1 inhibitor rapamycin abolished the nuclear translocation of Foxk1 in the presence or absence of insulin treatment in the alpha mouse liver 12 (AML12) cells [100]. Activation of mTORC1 can promote Foxk1 nuclear localization and suppression of autophagy in the nutrient-rich environment [101]. Inhibition of mTORC1 causes phosphorylation of Foxk1 by GSK3 and phosphorylated Foxk1 binds to the 14-3-3 interacting protein resulting in its cytosolic retention, further leading to derepression of autophagy genes [99,103]. Studies have shown that suppression of Foxk1 was shown to upregulate the expression of LC3 II and downregulate the expression of p62 [102]. These results further confirmed that suppression of Foxk1 expression can enhance autophagy in MGC803 and AGS cells [102]. However, the upregulation of Foxk1 phosphorylation by mTORC1 repression can be blocked via inhibition of GSK-3 using GSK-3 inhibitor, CHIR99021 and knockdown of GSK-3α and GSK-3β, suggesting the involvement of the GSK-3 signaling pathway in the regulation of FoxK1 [99].

## 7. Calcium Regulates the Activation of GSK-3β

The phosphorylation of GSK-3β is reported to be regulated by intercellular calcium levels [104,105,106]. Induction of intercellular calcium levels by the activation of ion-channel protein transient receptor potential cation channel subfamily V member 4 (TRPV4) triggers GSK-3β phosphorylation and inactivation [105]. Moreover, overexpression of transient receptor potential cation channel subfamily M member 4 (TRPM4) in LNCaP cells promoted GSK-3β phosphorylation at ser9 [106]. Induction of AKT1 phosphorylation and activation was also observed in the LNCaP cells overexpressing TRPM4 [106]. Epidermal growth factor (EGF) stimulates AKT activation through calcium and calmodulin [107]. The induction of AKT1 and GSK-3β phosphorylation by EGF can be suppressed by the inhibition of Ca^2+^/CaM signaling [106]. Furthermore, cells treated with TCN, an AKT inhibitor, showed that the phosphorylation of GSK-3β in EGF treated PC3 cells was reduced [106]. Studies have shown that rotenone, a pesticide associated with α-synuclein aggregation, induces intercellular calcium levels and suppresses AKT and GSK-3β phosphorylation [104,108]. In this study, they showed that BAPTA, a chelator of intracellular calcium, can alleviate the downregulation of AKT and GSK-3β phosphorylation induced by rotenone treatment [104]. Furthermore, rotenone caused impairment of autophagy, which can be prevented by inhibition of GSK-3β [104]. On the other hand, calcium also can regulate GSK-3β activity through calpain; calpain truncates the N-terminal regulatory domain of GSK-3β [109].

## 8. Conclusions

In this review, we discussed the mechanisms by which the GSK-3 signaling pathway regulates autophagy. The GSK-3 signaling pathway is implicated in the pathogenesis of several diseases such as neurodegenerative diseases and cancer [73,110]. Autophagy plays a vital role in maintaining cellular homeostasis and cell survival [33]. The GSK-3 signaling pathway was reported to regulate autophagy flux via the mTORC1, PKC and AKT signaling pathways [30,61,65,78,111]. mTORC1, known as a major autophagy regulator, is involved in the phosphorylation and inhibition of GSK-3β via its substrate, S6K [58,59]. GSK-3β also has the ability to regulate mTORC1 by phosphorylating Raptor on Ser859 directly [94]. Studies have also shown that GSK-3 can regulate mTORC1 by modulating the activity of TSC2 [12,95,96]. mTORC1 can not only regulate TFEB activity through GSK-3β-mediated TFEB phosphorylation but also regulate Foxk1 phosphorylation via GSK-3β [99]. ER stress is also known to induce GSK-3β activation and phosphorylate ULK1 leading to induction of autophagy [91]. On the other hand, several studies have also shown that inhibition of GSK-3β upregulates autophagy flux [11,12]. Moreover, GSK-3β can be also regulated by PKC and elF4A3 to trigger TFEB nuclear localization and activation [65,66]. In conclusion, the studies suggest that the GSK-3 signaling pathway regulates autophagy by modulating several signaling pathways in an mTORC1-dependent and independent manner.
